# Recurrence of odontogenic keratocysts and possible prognostic factors: Review of 455 patients

**DOI:** 10.4317/medoral.22827

**Published:** 2019-06-25

**Authors:** Nyimi-Bushabu Fidele, Zheng Yueyu, Yifang Zhao, Wu Tianfu, Jinyuan Liu, Yanfang Sun, Bing Liu

**Affiliations:** 1 MD. Resident. Department of Oral Maxillofacial Head and Neck Oncology Surgery, School, and Hospital of Stomatology, Wuhan University, Wuhan, China; Wuhan 430079, China; 2 DDS, MSc .Professor. Department of Oral Maxillofacial Head and Neck Oncology Surgery, School, and Hospital of Stomatology, Wuhan University, Wuhan, China; Wuhan 430079, China. The State Key Laboratory Breeding Base of Basic Science of Stomatology & Key Laboratory of Oral Biomedicine Ministry of Education, School & Hospital of Stomatology, Wuhan University, Wuhan 430079, China; 3 Ph.D. Resident. Department of Oral Maxillofacial Head and Neck Oncology Surgery, School, and Hospital of Stomatology, Wuhan University, Wuhan, China; Wuhan 430079, China; 4 Ph.D Associate Professor. Department of Oral Maxillofacial Head and Neck Oncology Surgery, School, and Hospital of Stomatology, Wuhan University, Wuhan, China; Wuhan 430079, China. The State Key Laboratory Breeding Base of Basic Science of Stomatology & Key Laboratory of Oral Biomedicine Ministry of Education, School & Hospital of Stomatology, Wuhan University, Wuhan 430079, China

## Abstract

**Background:**

To describe epidemiological features of 565 Chinese patients with odontogenic keratocysts (OKC), to investigate possible prognostic factors related to recurrence, and to analyse features of recurrent OKC (rOKC).

**Material and Methods:**

A retrospective chart review of 565 cases of OKC treated between 2003 and 2015 was undertaken. The probability of recurrence related to prognostic factors including large size, cortical perforation combined with involved teeth in the lumen of the cyst, inflammation, sites of the involved lesion, sex, and daughter cyst variables were analysed. The subsequent relapse of each OKC was compared.

**Results:**

Patients ranged in age from 7 to 81 years (mean age, 28.4 years) and, of those affected, 66.9% were male and 33.1% were female. Mandibular OKC occurred in 63.01% and 36.99% occurred in the maxilla, 80.53% of patients had non-rOKC, 10.44% rOKC, and 9.03% had multiple OKC lesions. Enucleation with preservation of the involved teeth in the cystic lesion combined with cortical perforation was statistically associated with high recurrence rate, as were daughter cysts, and multilocular lesions. The number of recurrences and the average time (in years) to relapse decreased from the first relapse of OKC to the third relapse, and the difference was significant (*P*<.05).

**Conclusions:**

Preservation of the involved teeth combined with cortical perforation appeared to be a potential prognostic factor associated with high recurrence. The follow-up evaluation period for rOKC with ≥ 2 previous treatments should be shorter than for first-time rOKC. The decreasing average duration (years postoperatively) to relapse was related to the number of rOKCs, timing of relapse, and rOKC type.

** Key words:**Keratocystic odontogenic tumor, risk of recurrence, involved teeth and cortical perforation, relapse, epidemiological features.

## Introduction

The odontogenic keratocyst (OKC) is a distinctive entity, known for aggressive behaviour and high recurrence rates. OKCs are generally solitary, although they can present with multiple lesions, and are usually associated with nevoid basal cell carcinoma syndrome (NBCCS) (1); malignant transformation has been reported (2). Various studies have reported recurrence rates of up to 62.5% (3-6), with the majority of recurrences arising within the ﬁrst 5 years following initial treatment (7,8). Although there are numerous possible explanations for the frequent recurrence of OKCs, there is considerable debate regarding the exact reason. Clinicohistopathological studies and those focused on treatment methods related to recurrence have suggested that recurrence was due to operative treatment methods, rather than clinicopathological factors (5,9,10). On the other hand, various recent investigations have been performed to link the recurrence of OKC to clinicopathological factors such as large size, cortical perforation, tooth involvement in the lumen of the cyst, and daughter cysts (11-13). The findings appear to suggest that these prognostic factors may predict a high risk for recurrence. However, such studies are few in number, and the outcomes should be interpreted cautiously due to the potentially limited number of patients examined. In addition, there is currently no detailed information about the clinical and histological features of Chinese patients with OKC. Accordingly, the objectives of the present study were as follows: to describe epidemiological features of 565 Chinese patients with OKC (recurrent OKC [rOKC], nonrecurrent OKC[nrOKC], and multiple OKC, and association with NBCCS; to investigate the risk for recurrence of OKC in relation to prognostic factors; and to analyse the features of OKC in each postoperative relapse to improve the follow-up evaluation.

## Material and Methods

A retrospective chart review of 565 Chinese patients with 663 OKCs based on diagnostic criteria outlined in the 2017 World Health Organization classification (14) was performed. Data were collected from patients treated between 2003 and 2015 at the Department of Oral and Maxillofacial Surgery, in Wuhan Hospital, and School of Stomatology. Patients were excluded if follow-up occurred < 2 year following initial treatment, or if their histopathological diagnosis was an orthokeratinized variant, or rOKC treated at another hospital without details of the primary lesion. Patient sex, site of involvement, complaints, teeth involved in the cystic lesion, size, cortical perforation, radiographic appearance, treatment modality, and recurrence were evaluated. The lesions were measured in centimetres on the panoramic radiographs taken before surgical treatment along the major and minor axis of the cyst. Treatment methods included marsupialization combined with enucleation, enucleation, and radical resection. Enucleation and marsupialization were usually performed using intraoral approach. Teeth involved in the margins of the cyst were treated by extraction so as to remove any doubt of leaving pathological tissue behind, or if the roots extended into the cyst lumina and interfered with the complete removal of the cyst wall. The treatment procedures were explained and discussed with the patients, before the ﬁnal decision. Some patients (n=56) were not comfortable with the tooth extraction protocol and favoured the risk for recurrence over losing vital teeth. Histopathological factors (such as daughter cysts and inflammation) were analysed. The microscopic slides of the patients were reviewed by a panel of three oral pathology specialists to confirm a diagnosis of OKC and to evaluate histopathological characteristics. Inflammation was considered to be significant if it altered the epithelial lining of the lesion or densely covered at least one-quarter of the cyst wall. The probability of recurrence was evaluated in relation to prognostic factors.

Among the 565 patients, 110 were excluded from the study for following reasons: failure to attend follow-up (n=89); data records incomplete for review (n=7); presence of orthokeratinized variant (n=8); rOKC from other hospitals (n=6). A total of 455 patients was finally reviewed, of which 351 were nrOKC, 54 were rOKC and 50 had multiple OKC lesions. Postoperative follow-up evaluation, as a means of estimation of the frequency of recurrence was performed for nrOKC. When possible, all patients were annually reviewed radiologically and clinically after the ﬁrst re-examination 6 months after resection of the lesion or contacted by mail for failure to return. The disease-free time or relapse period was defined as the time between surgery and the detection of recurrence. Surgical management of recurrence was classified as enucleation combined with adjuvant therapy including (peripheral ostectomy or application of Carnoy’s solution), and the resection. Each recurrent lesion was also reviewed in relation to previously described diagnostic criteria (14). The judgment criterion was the presence or absence of recurrence. Survival curves were exhibited using Kaplan-Meier method and log-rank test. Differences in clinicopathological prognostic factors and recurrence were evaluated by the chi-squared test or Fisher’s exact test. Univariates and multivariate analysis were performed to identify the prognostic factors associated to the recurrence. The cox proportional hazards model for time-dependent variables was used to calculate hazard ratio estimates and corresponding 95% conﬁdence interval (CI) as estimates of hazard ratio (HR) for recurrent potential. The prognostic factors that were associated or not to the recurrence in the univariate analysis, were included in the cox model to determine the independent factors. Statistical significance was set at 5%.

## Results

**Figure 1:**
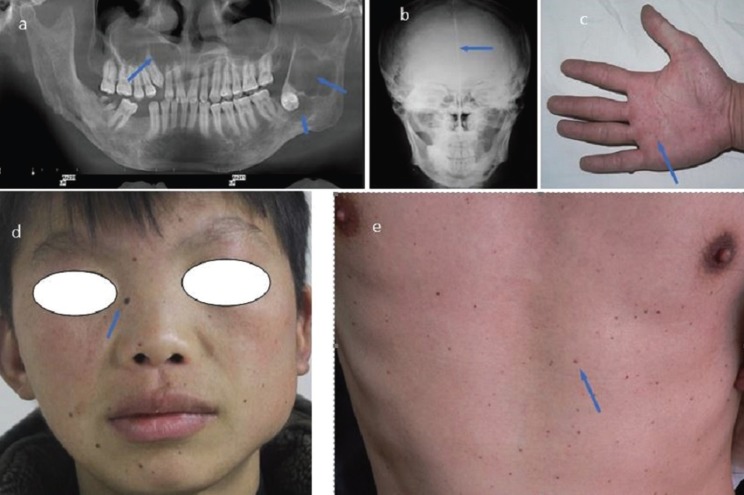
Some of the clinical manifestations of nevoid basal cell carcinoma syndrome. Presence of multiples synchronous odontogenic keratocysts in the maxilla and mandible with unilocular pattern involving third impacted molar (a). Antero-posterior skull radiograph revealing calcification of the falx cerebri (b), multiple palmar pits (c). Multiple cutaneous nevi located in all facial and thoracoabdominal region (d, e).

Patient age varied between 7 and 81 years. Demographic information and clinical characteristics of the 565 patients with OKCs are summarized in  Table 1. Of the OKCs, 457 (68.92%) were unilocular radiolucent lesions and 206 (31.07%) were multilocular. Many patients presented as painless (56.11% [ n=317]), while others had various complaints including swelling/pain (22.48% [n=127]), pus discharge (8.49% [n=48]), and paraesthesia (1.06% [n=6]). Incidental radiographic findings of the condition were observed in 11.85% (n=67). With regard to histopathological features, no significant difference was observed among those with nrOKC, rOKC and OKC with multiple lesions. However, the daughter cysts variable was significantly higher for OKC with multiple lesions than for nrOKC or rOKC. In 19 (3.36%) patients, the diagnosis of NBCCS was confirmed, which was characterized by the presence of multiple OKCs, multiple cutaneous nevi, multiple palmar pits, and falx cerebri (Fig. 1). Among 50 patients with multiple lesions, 6 presented with a single OKC, 11 had 2 OKCs, 18 had 3 OKCs, 9 had 4 OKCs, and 6 had 5 OKCs. However, despite these multiple lesions (51-19), it was insufficient for the diagnosis of NBCCS.

**Table 1:**
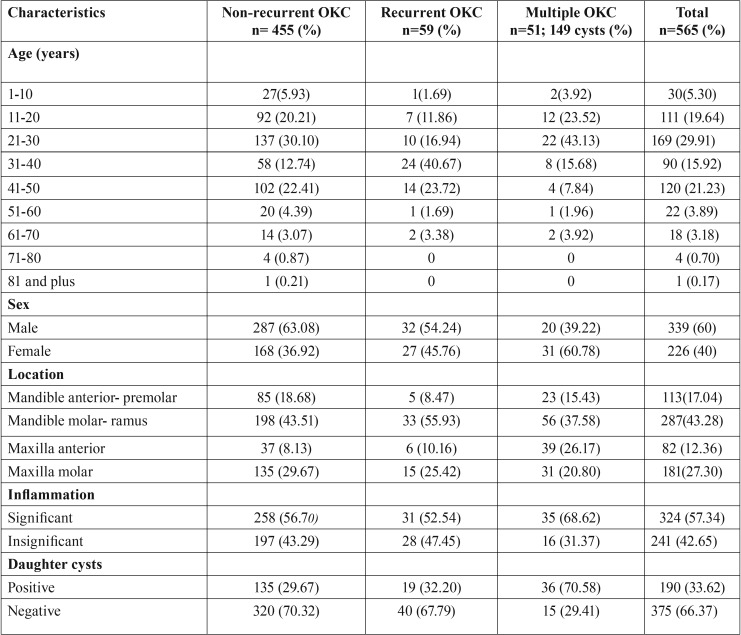
Demographic and clinical characteristics of 565 patients with 663 odontogenic keratocysts.

**Figure 2:**
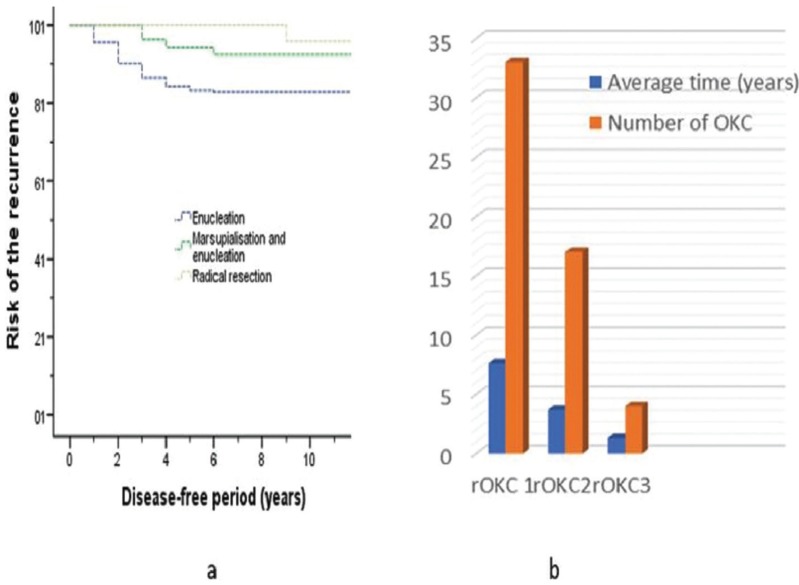
The cumulative risk of recurrence.(a) Kaplan–Meier curves according to surgical methods for the nonrecurrent OKC. (b) Recurrent odontogenic keratocyst type according to number and average time.

**Figure 3:**
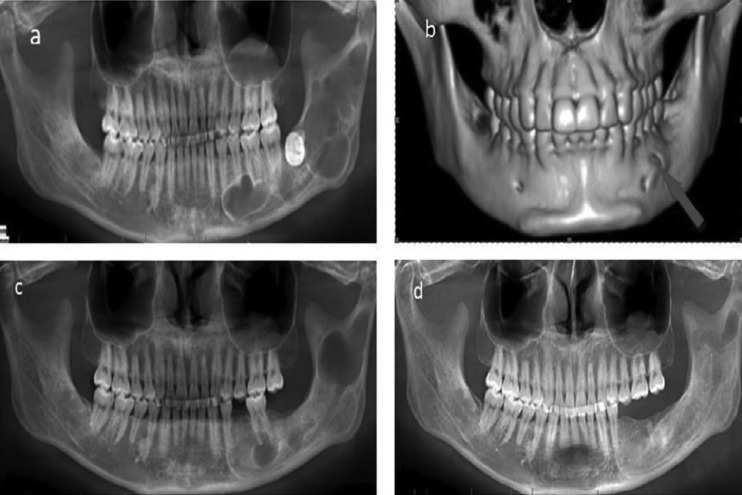
Recurrence of odontogenic keratocyst (OKC) in relation to cortical perforation and preservation of the involved tooth in the margin of the lesion. Primary OKC before enucleation (a), the presence of cortical perforation (blue arrow, b). Recurrence of OKC 1 postoperative year after enucleation with preservation of involved left first molar (c), and therapeutic monitoring 2 years after a second enucleation with the extraction of a left mandibular first molar (d).

Cyst size varied between 1cm and 13cm, with an average size of 4.3cm. The recurrence rate was 15.09% (53/351) following a postoperative observation period of 2 to 12 years (average, 5.2 years). Enucleation was the most commonly used treatment method (274/351), and most associated with a high risk for recurrence (48/274 [17.52%]). Marsupialization combined with enucleation had lower recurrence, as did resection (4/53 [7.5%] and 1/24 [4.17%] respectively). Fig. 2a shows a difference in recurrence patterns between the three types of treatment as displayed by the graphics of the Kaplan-Meier analysis (*P*=.021). The probability of recurrence related to prognostic factors was evaluated, and the results are summarized in  Table 2. Patients treated with enucleation, with preservation of involved teeth in contact with the margins of the cyst (n=56), was significantly associated with a high recurrence rate compared with patients treated with enucleation with extraction of involved teeth (HR 4.044 [95% CI 1.816-9.004]; *P*=.001). Most importantly, 19 patients had involved teeth combined with cortical perforation (Fig. 3), and recurrence was found in 100% of these cases. However, among the 37 patients who had involved teeth without cortical perforation, recurrence occurred in 62.16% (23/37). In addition, daughter cysts and multilocular lesions were also significantly related to recurrence ([HR 5.452 [95% CI 1.860-15.974], *P*=.002), and HR 1.852 [95% CI 1.064-3.223], *P*=.029). In the univariate analysis as well as in the multivariate analysis; sex, sites of involvement and inflammation covariables were not associated with recurrence. The size of lesion was significantly associated with high recurrence rates by using the univariate analysis (*P*=.013), however, the feature was not determined as independent factor in the multivariate analysis.

**Table 2:**
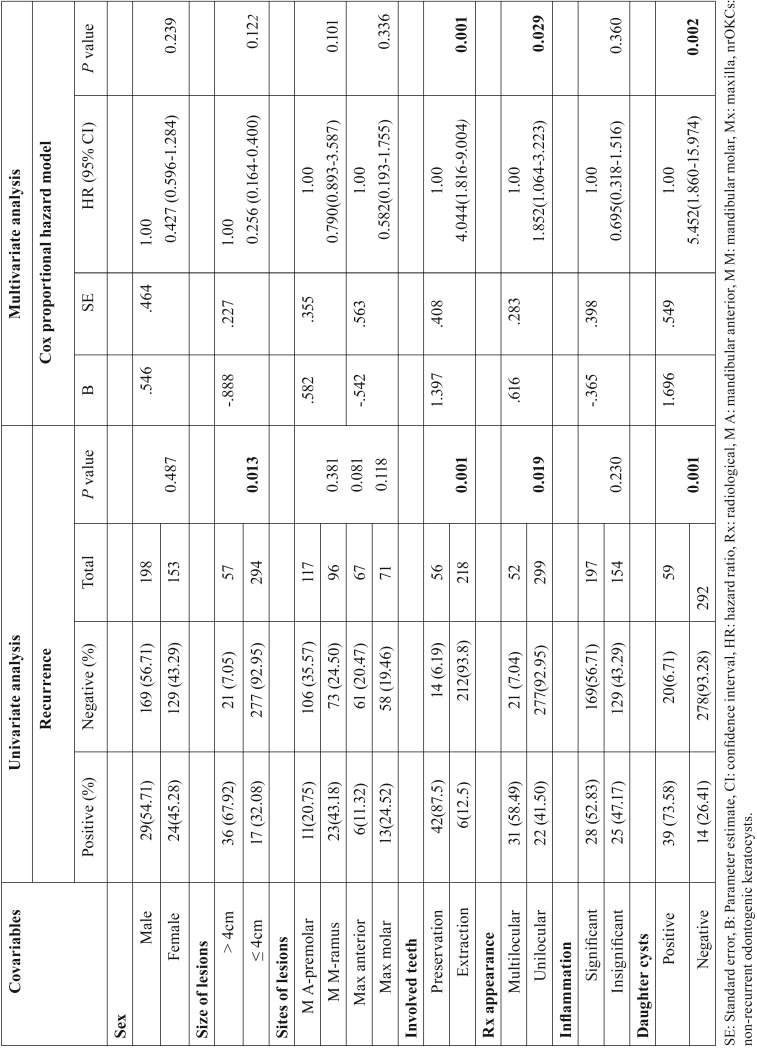
The probability of recurrence related to clinicopathological prognostic factors in 351 non-recurrent odontogenic keratocysts.

 Table 3,  Table 3 continue summarizes the features of rOKC. The most commonly affected sites for rOKC were the mandibular molar-ramus area (57.41% [31/54]) and maxillary molar area (24.07% [13/54]). More than one-half of rOKC patients (61.1% [n=33]) had undergone 1 previous surgery. The time interval from the initial treatment to a period of relapse varied from 6 months to 40 years (average duration, 7.6 years). The recurrences were treated using enucleation combined with peripheral ostectomy or use of application of Carnoy’s solution, and by segmental resection ( Table 3). Other rOKC patients had undergone 2 previous surgeries (31.5% [n=17]). The time interval from the second treatment to the second relapse varied between 1 and 14 years (average duration, 3.8 years). A few rOKC patients (7.4% [n=4]) underwent 3 previous surgeries. The time period from the third surgery to the third relapse ranged from 1 to 2 years (average duration; 1.3 years). The treatment of recurrence in both was enucleation with subsequent adjuvant therapy. The majority of recurrences occurred within the first 5 years postoperatively, with decreasing incidence from 6 to 41 years postoperatively. The number of recurrences and the average year of relapse continually decreasing from the first rOKC (rOKC1 or recurrent OKC who had undergone 1 previous surgery) to third rOKC (rOKC3 or recurrent OKC who had undergone 3 previous surgeries), and the difference was statistically significant, (*P*<.05) (Fig. 2b).

**Table 3:**
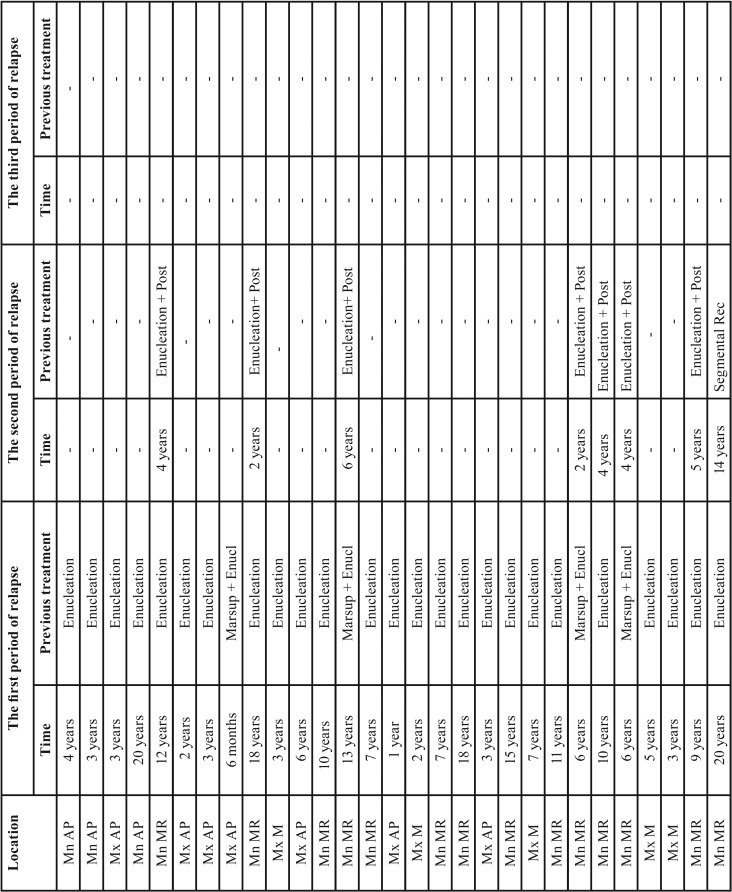
Characteristics of recurrent odontogenic keratocysts.

**Table 3 continue:**
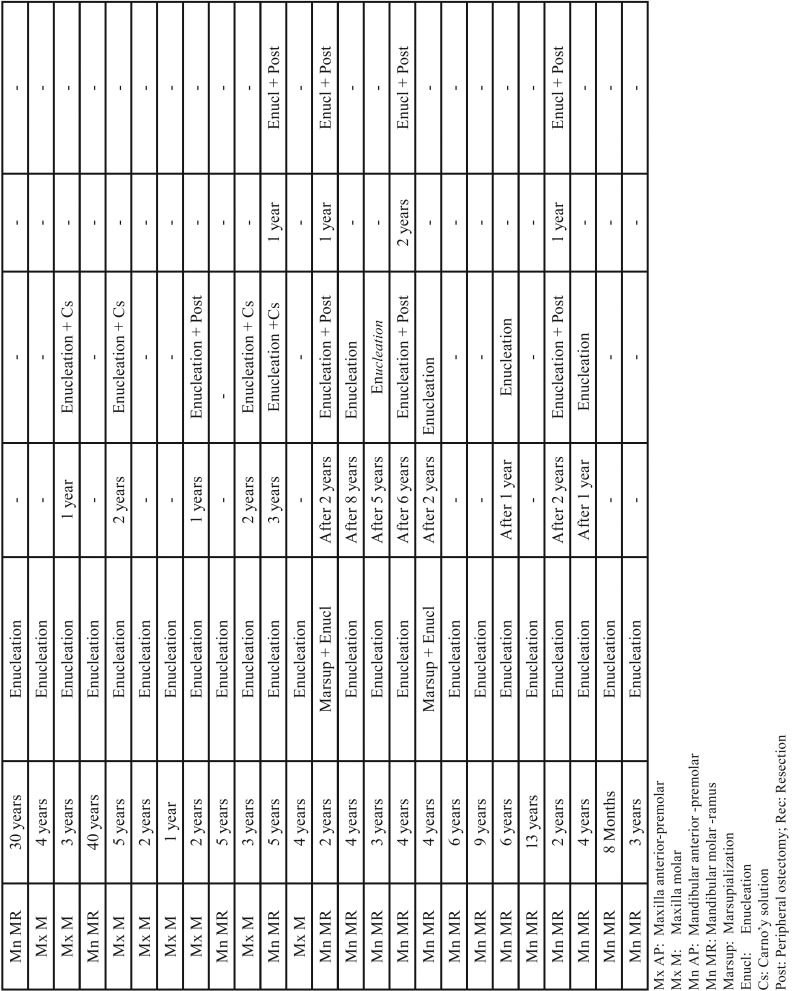
Characteristics of recurrent odontogenic keratocysts.

## Discussion

OKC remains a lesion that exhibits ambiguous behaviour. Herein, we reported detailed epidemiological features of OKC, and the risk for recurrence related to prognostic factors and the features of rOKCs. This is one of the first of a large series aiming to provide a detailed view of epidemiological and prognostic factors. No consensus was found in the literature regarding the sex distribution of OKC. Some studies have reported a male predominance (15,16), as in the present study, while others have not (11,17). Patient age at diagnosis (second-third decades of life) and the most common sites of affected by OKC (mandibular molar-ramus) were consistent with previous findings (16,18). Patient complaints were not different, regardless of whether they had rOKC, nrOKC or multiple lesions. Patients most commonly presented as painless or with swelling/pain (19,20). Multiple OKCs in this study, present a weak expression of the syndrome. Our results are in consistent with the previous reports (15,19). OKCs associated with NBCCS have been linked to mutations in the patched tumor suppressor. In the absence of other features of NBCCS, partial expression of the gene or the multifocal nature of OKCs may result in the occurrence of only multiple OKCs yet without evidence of clinical manifestations of the syndrome. Therefore, their clinical manifestations may remain hidden in the earlier years of life (21). Thus, maybe rise the possibility of a weak expression of NBCCS. Additionally, patients with NBCCS differs from ethnic groups due to variation in penetration and expression of different mutations within the Patched (PTCH) gene and environmental factors (22). NBCCS as an autosomal dominant inherited condition, exhibits the high variable expressivity of clinical features (23-25). However, the most common clinical features of NBCCS are multiple basal cell carcinomas, multiple OKCs and skeletal anomalies. OKCs are one of the most constant clinical features in the development of NBCCS, and one of the characteristic features of this syndrome, which may appear in very early life that can lead to its recognition at that time (26). According to Kimonis et al., the above-mentioned clinical features do not develop until the teenage years, therefore, it is difficult to diagnosis the affected child with this syndrome (24). The observed palmar and plantar pits, palatal abnormalities, macrocephaly, and bridged sella are more indicative for the diagnosis of NBCCS in yang patients than jaw cysts, and multiple basal cell carcinomas (25). In the present study, the most prevalent clinicals features of NBCCS was multiple OKCs, multiple cutaneous nevi, multiple palmar pits, and falx cerebri. No sex predilection for multiples OKCs have been reported. The present study combined with others reports suggested female predilection (18,27), while others investigators suggested male predilection or equal gender distribution (19,28).

Various studies that have evaluated prognostic factors related to recurrence of OKC have reported an association with a high risk for recurrence. Although these ﬁndings may be of some value in predicting the risk for recurrence, results of these investigations must be considered with caution due to the limited number of patients examined (11,12). The observed recurrence rate in the present study was 15.09%. Although an association between sex and rOKC has previously been reported (29), the present study in addition to others, did not confirm this (6,30). The relationship between the site of involvement of OKC and recurrence is controversial (13,31). In our series, the sites of OKC were not statistically related to recurrence. However, the posterior mandibular or maxillary regions were the most high-risk location for recurrence. The anatomical characteristics of thin cortex, spongy nature of the maxilla with multiple cavities, difficulty with access impeding complete removal of the entire cyst, close relationship of the margins of the cyst to vital structures with attempted conservative treatment methods as enucleation, and the high occurrence of the frequent cortical perforation and firm adhesion of the cysts to the overlying mucosa, are possible explanations for the high-risk location of recurrence.

To the best of our knowledge, no previous studies have demonstrated the combination of cortical perforation and involved teeth treated conservatively compared with the conservative treatment of involved teeth without cortical perforation as a significant factor in the increased risk for recurrence. It is known that the close relationship of the cyst with the dental root, or an attempt to save vital involved teeth with enucleation alone during the operation, usually lead to the incomplete eradication of the lesion with a significant recurrence rate (11,12). More importantly, the present study found that recurrence was highly associated with involved teeth preserved in the cyst combined with cortical perforation. To extract or preserve the involved teeth in the cyst remains a dilemma that is usually encountered by surgeons. Extraction of supernumerary teeth, impacted teeth, teeth without function and those of recurrent cases is, no doubt, one of the necessary measures. However, in other situations, the treatment of involved teeth remains unclear. Previous studies have recommended removal of teeth involved in the cyst if there is any doubt of leaving pathological tissue behind (12). Although enucleation is the gold standard for OKC treatment, it is not sufficient to reduce the risk for recurrence for patients with high-risk factors. Therefore, effective elimination of high-risk factors should be carefully considered. We suggest that the treatment methods should aim to avoid occlusion complications, such as enucleation with adjuvant therapies such as peripheral ostectomy, apicoectomy, root canal treatment, application of use Carnoy’s solution and superperiosteal dissection, or marsupialization followed enucleation. This aims to balance the preservation of functional teeth, bone structure, and reduction of recurrence. Kolokythas et al (29) reported that no recurrence was found in patients treated with combined enucleation with peripheral ostectomy and those treated using surgical resection in the same group. It has been unexpectedly observed that when the preservation of involved teeth was combined with cortical perforation, recurrence was as high as 100%. However, the relationship between this particular situation and high recurrence rates is unknown; therefore, it would be premature to comment in the absence of further empirical data. Nevertheless, the result may be of particular importance in drawing attention to the treatment methods that should be used.

In addition, it is known that large OKC linings are very thin and fragile, and are more prone to recur than smaller lesions, which are easier to enucleate in one piece. The correlation between size and OKC recurrence was examined, and cysts > 4 cm demonstrated a significantly high recurrence rate compared with size < 4 cm in the univariate analysis (*P*=.013). This finding was consistent with other reports (6,30); however, size was not associated with a high risk for recurrence (HR 0.256 [95% CI 0.164-0.400]; *P*=0.122). Multilocular lesions and daughter cyst formation were both associated with a higher recurrence rate, a finding consistent with previous studies (19,32). However, Naruse *et al.* (12) and Kaczmarzyk *et al.* (13) did not find any association between daughter cysts and recurrence rate. The discrepancy may be due to variations in the number of patients examined or the length of the follow-up period.

OKCs recur mostly within 5-7 years of the initial surgery, and a significant number of recurrences may not manifest until ≥10 years after the original surgical procedure. Although recurrence within 30-40 years has been rarely reported (33), the present study found 2 rOKCs within 30 and 40 years, respectively, after initial treatment. The incidence of recurrence greatly increased within the first five years after operation and decreased from the first 10 years to 40 years postoperatively. This suggest that, as the number of postoperative years increases, the risk for disease relapse decreases, and that follow-up evaluation should be focused on the first 5-7 years after initial treatment, with annual examination to complete years of follow-up. In addition, the number of recurrences and the average time (in years) for relapse of OKC are continually decreasing for first-time rOKC (i.e., recurrent OKC with 1 previous surgery) to third-time rOKC (i.e., recurrent OKC with 3 previous surgeries); this means that the decreasing average year of relapse is related to the number of rOKCs, the timing of a relapse and the rOKC type. This finding, never previously reported, suggests that the follow-up evaluation should be even closer than rOKCs with 1 previous surgery. Because the presence of epithelial budding and islands close to the overlying oral mucosa is highest in rOKCs, those lesions should be treated with more radical surgery (such as enucleation combined with peripheral ostectomy, or use of Carnoy’s solution, or en bloc resection) than previous treatments (34,35). These treatment modalities aim to eliminate epithelial islands and microcysts in the peripheral bone and, moreover, decrease recurrence rates. In addition, the fact that OKCs can recur as multiple or solitary lesion, may make the assessment of recurrence difﬁcult, mostly when it is associated with NBCCS (8). Thus, multiple cysts are more likely recur, especially in younger patients and, therefore, close follow-up is essential.

## Conclusions

Findings regarding epidemiological features in the present study were similar to those published in earlier reports.

The preservation of involved teeth combined with cortical perforation appear to be associated with the highest likelihood of recurrence. The decreasing average duration (years postoperatively) to relapse was related to the number rOKCs, the timing of a relapse and rOKC type. The follow-up evaluation period for rOKC with 2 previous treatments should be shorter than for first -time rOKCs.
